# *Acinetobacter* Peritoneal Dialysis Peritonitis: A Changing Landscape over Time

**DOI:** 10.1371/journal.pone.0110315

**Published:** 2014-10-14

**Authors:** Chia-Ter Chao, Szu-Ying Lee, Wei-Shun Yang, Huei-Wen Chen, Cheng-Chung Fang, Chung-Jen Yen, Chih-Kang Chiang, Kuan-Yu Hung, Jenq-Wen Huang

**Affiliations:** 1 Division of Nephrology, Department of Internal Medicine, National Taiwan University Hospital Jin-Shan Branch, New Taipei City, Taiwan; 2 Graduate Institute of Toxicology, National Taiwan University, Taipei, Taiwan; 3 Division of Nephrology, Department of Internal Medicine, National Taiwan University Hospital Yun-Lin Branch, Yun-Lin County, Taiwan; 4 Division of Nephrology, Department of Internal Medicine, National Taiwan University Hospital Hsin-Chu Branch, Hsin-Chu County, Taiwan; 5 Division of Nephrology, Department of Internal Medicine, National Taiwan University Hospital, Taipei, Taiwan; 6 Department of Emergency Medicine, National Taiwan University Hospital, Taipei, Taiwan; 7 Department of Geriatrics and Gerontology, National Taiwan University Hospital, Taipei, Taiwan; 8 Department of Integrative diagnostics and Therapeutics, National Taiwan University Hospital, Taipei, Taiwan; University of Malaya, Malaysia

## Abstract

**Background:**

*Acinetobacter* species are assuming an increasingly important role in modern medicine, with their persistent presence in health-care settings and antibiotic resistance. However, clinical reports addressing this issue in patients with peritoneal dialysis (PD) peritonitis are rare.

**Methods:**

All PD peritonitis episodes caused by *Acinetobacter* that occurred between 1985 and 2012 at a single centre were retrospectively reviewed. Clinical features, microbiological data, and outcomes were analysed, with stratifications based upon temporal periods (before and after 2000).

**Results:**

*Acinetobacter* species were responsible for 26 PD peritonitis episodes (3.5% of all episodes) in 25 patients. *A. baumannii* was the most common pathogen (54%), followed by *A. iwoffii* (35%), with the former being predominant after 2000. Significantly more episodes resulted from breaks in exchange sterility after 2000, while those from exit site infections decreased (*P* = 0.01). The interval between the last and current peritonitis episodes lengthened significantly after 2000 (5 vs. 13.6 months; *P* = 0.05). All the isolates were susceptible to cefepime, fluoroquinolone, and aminoglycosides, with a low ceftazidime resistance rate (16%). Nearly half of the patients (46%) required hospitalisation for their *Acinetobacter* PD-associated peritonitis, and 27% required an antibiotic switch. The overall outcome was fair, with no mortality and a 12% technique failure rate, without obvious interval differences.

**Conclusions:**

The temporal change in the microbiology and origin of *Acinetobacter* PD-associated peritonitis in our cohort suggested an important evolutional trend. Appropriate measures, including technique re-education and sterility maintenance, should be taken to decrease the *Acinetobacter* peritonitis incidence in PD patients.

## Introduction

The utilisation of peritoneal dialysis (PD) in end-stage renal disease (ESRD) has been long purported for its economical advantage and similar effects on patient outcomes compared with haemodialysis (HD) [1], [2]. However, the underuse of PD in most countries could be attributable to several factors, including the infectious complications [1], [3]. Among these complications, PD-associated peritonitis is the most dreadful, accounting for 8% to 21% of infection-related mortality and 30% of technique failures [4], [5]. Gram-negative bacteria (GNB) cause 25% to 40% of PD peritonitis episodes worldwide, secondary to gram-positive bacteria, and the number is still increasing [6], [7].

Among GNB peritonitis, the acronym SPICE (*Serratia*, *Providencia*, indole-positive *Proteus/Acinetobacter/Morganella*, *Citrobacter*, *Enterobacter*, or *Hafnia*) denotes a spectrum of organisms with an intrinsic tendency for antibiotic resistance [8]. These pathogens demonstrate inducible beta-lactamase during initial therapy, potentially leading to empirical regimen failure, prolonged peritoneal damage, and worse outcomes [9]. In the ANZDATA registry, the SPICE group of pathogens accounts for 26.9% of all non-*Pseudomonas* GNB PD-associated peritonitis, and *Acinetobacter* species are responsible for <20% of these cases [10], [11]. Intuitively thinking, patients with *Acinetobacter* PD-associated peritonitis might have poorer prognoses compared with the other Enterobacteriaceae members, owing to their high antibiotic resistance rates. However, past reports suggested just the opposite, with technique failure rates of 9% [12] and 9.2% [13] in several studies. In addition, several researchers proposed that *Acinetobacter* peritonitis might occur with an immunocompromised status [12], [14]. These reports were all published nearly 2 decades ago, and the temporal changes of clinical characteristics, microbiologic features, and outcomes of patients with *Acinetobacter* PD peritonitis are unknown. Utilising a longitudinal PD registry of more than 2 decades in a single centre, we conducted the present study to explore the transition of these parameters over different time periods.

## Subjects and Methods

### Ethical considerations

The ethics committee of the NTUH approved the current study (No. 201212165RINC). The local institutional review board did not mandate patient consent, as no interventions were performed and patient privacy was not breached. The local institutional review board waived the need for written informed consent from the participants.

### Study design and setting

In 1985, the National Taiwan University Hospital (NTUH) PD program was established [9], [15]. Patients with ESRD who underwent PD for >3 months were eligible for the present study. Patients with episodes of culture-confirmed *Acinetobacter* species PD peritonitis between 1985 and 2012 were selected. Peritonitis was diagnosed according to the presence of symptomatology and cloudy effluent, with a white blood cell count >100/µL and neutrophil percentage >50% [5].

### Clinical data collection

All enrolees’ demographic profiles, including age, sex, and comorbidities (diabetes mellitus [DM], heart failure [HF], and autoimmune diseases), were reviewed [16], [17]. The aetiologies of their ESRD were also documented. Through a chart review, patients’ medications, including steroid, immunosuppressant, and antibiotic use, within 1 month before the selected peritonitis episodes were recorded, along with laboratory data.

For each peritonitis episode caused by *Acinetobacter*, we documented the PD vintage and modality (continuous ambulatory peritoneal dialysis [CAPD] or automated peritoneal dialysis [APD]) and the initial symptoms. Laboratory data during the peritonitis episodes were obtained. Pathogen species and the corresponding antibiogram were identified. The presumptive origins of each peritonitis episode were divided into a break in sterility during the exchange procedure, gastrointestinal (GI) microflora transmural migration, exit site infection/tunnel infection, or undetermined, according to past reports [18], [19]. Empirical antibiotic regimens for PD peritonitis in our institute were as follows: first-generation cephalosporin with aminoglycoside before 1998 and first-generation cephalosporin with third-generation cephalosporin after 1998, according to the recommendations of the International Society for Peritoneal Dialysis (ISPD), unless otherwise indicated [5], [9], [15], [20], [21].

### Outcome variables

The outcome measures in the present study consisted of primary response, antibiotic switch (secondary response), relapse peritonitis, repeat peritonitis, Tenckhoff catheter removal, and patient death. The primary response was defined as symptomatic improvement of the patient within 3 days of receiving empirical antibiotics, accompanied by effluent leukocyte count <100/µL. The secondary response was defined as a response to the second-line antibiotics when effluent did not turn clear after first-line antibiotic use [9], [15]. If an antibiotic switch occurred, the duration of empirical antibiotic use was also recorded. Repeat peritonitis was defined as peritonitis recurring 4 weeks after the previous episode, whereas relapse peritonitis was defined as peritonitis recurring within 4 weeks after the treatment of the previous episode, both involving the same pathogen [5], [21]. Peritonitis-related death was considered if the patient’s death occurred within 1 month of the peritonitis episode and was attributable to peritonitis [9], [15].

## Results

During the study period, 369 of 1278 chronic PD patients developed 744 episodes of PD-related peritonitis over 40,499 patient-months, with an overall peritonitis incidence of 1 per 54 patient-months (before 2000, 1 per 37 patient-months; after 2000, 1 per 64 patient-months). Among the 744 episodes of PD-related peritonitis, 25 patients developed 26 peritonitis episodes from *Acinetobacter* species (3.5% of all episodes), with an incidence of 1 per 1558 patient-months. One patient developed 2 separate episodes of *Acinetobacter* peritonitis, and the remaining patients each experienced 1 episode. To delineate the temporal changes of their clinical features, we divided the *Acinetobacter* peritonitis cohort into 2 phases, those that occurred before 2000 and those that occurred during or after 2000.

### Clinical features of Acinetobacter PD-associated peritonitis

The demographic profiles and clinical characteristics of the patients who developed PD-related peritonitis caused by *Acinetobacter* are listed in Table 1, according to the time of onset. Overall, the mean age and PD vintage of the patients with *Acinetobacter* peritonitis were 52 years and 29 months, respectively, without differences between phases. Three-fourths of the patients chose CAPD as their dialysis modality. One-third of *Acinetobacter* peritonitis patients had DM, but only 12% of patients had HF.

**Table 1:** Baseline features of patients with *Acinetobacter* PD-associated peritonitis.

*Characteristics*	*Total (n = 26)*	*Before 2000* *(n = 12)*	*After 2000* *(n = 14)*	*P* *value* *
**Incidence (one episode per patient-months)**	1558	805	2053	<0.01
Age (years)	52 (23–84)	48 (37–71)	55 (23–84)	0.11
Gender (male %)	11 (42)	4 (33)	7 (50)	0.32
Vintage (months)	29 (1–80)	27 (1–80)	31 (3–60)	0.45
Modality (CAPD %)	19 (73)	10 (83)	9 (64)	0.29
**Comorbidities**				
DM	8 (31)	2 (16)	6 (43)	0.12
Heart failure	3 (12)	3 (25)	0 (0)	0.08
Autoimmune disorders	2 (8)	1 (8)	1 (7)	0.96
**ESRD aetiology**				0.15
DM	8 (31)	2 (16)	6 (43)	
CGN	8 (31)	4 (33)	4 (29)	
Hypertension	1 (4)	0 (0)	1 (7)	
Lupus	1 (4)	1 (8)	0 (0)	
Congenital renal dysplasia	1 (4)	0 (0)	1 (7)	
Unknown	7 (23)	5 (42)	2 (7)	
**Laboratory data**				
Albumin (mg/dL)	3.7 (2.5–4.2)	3.6 (2.5–4.2)	3.7 (2.6–4.2)	0.36
Hemoglobin (g/dL)	9.6 (6.3–13.8)	8.4 (6.3–13.8)	10.6 (8.4–11.7)	0.01
Creatinine (mg/dL)	11 (7.1–16.1)	12.2 (8.8–16.1)	10 (7.1–14.4)	0.07
Total cholesterol (mg/dL)	187.8 (113–321)	198 (124–321)	179.1 (113–267)	0.38

*Comparison between before-2000 group and after-2000 group.

Continuous variables are expressed in mean (ranges), while categorical variables are expressed in number (percentage in parentheses).

Abbreviations: CAPD, continuous ambulatory peritoneal dialysis; CGN, chronic glomerulonephritis; DM, diabetes mellitus; ESRD, end-stage renal disease.

The most common aetiologies of ESRD in the *Acinetobacter* peritonitis patients included DM (31%) and chronic glomerulonephritis (31%), followed by lupus (4%) and hypertension (4%). No significant difference was found between those that occurred before 2000 and those that occurred after 2000. Only 1 patient had undergone intra-abdominal surgery (cholecystitis with open cholecystectomy) before developing *Acinetobacter* peritonitis. Another patient received an antibiotic within the month before the current peritonitis episode (cephalexin for cellulitis for 1 week), and still another lupus patient was receiving prednisolone 10 mg daily.

**Figure 1:**
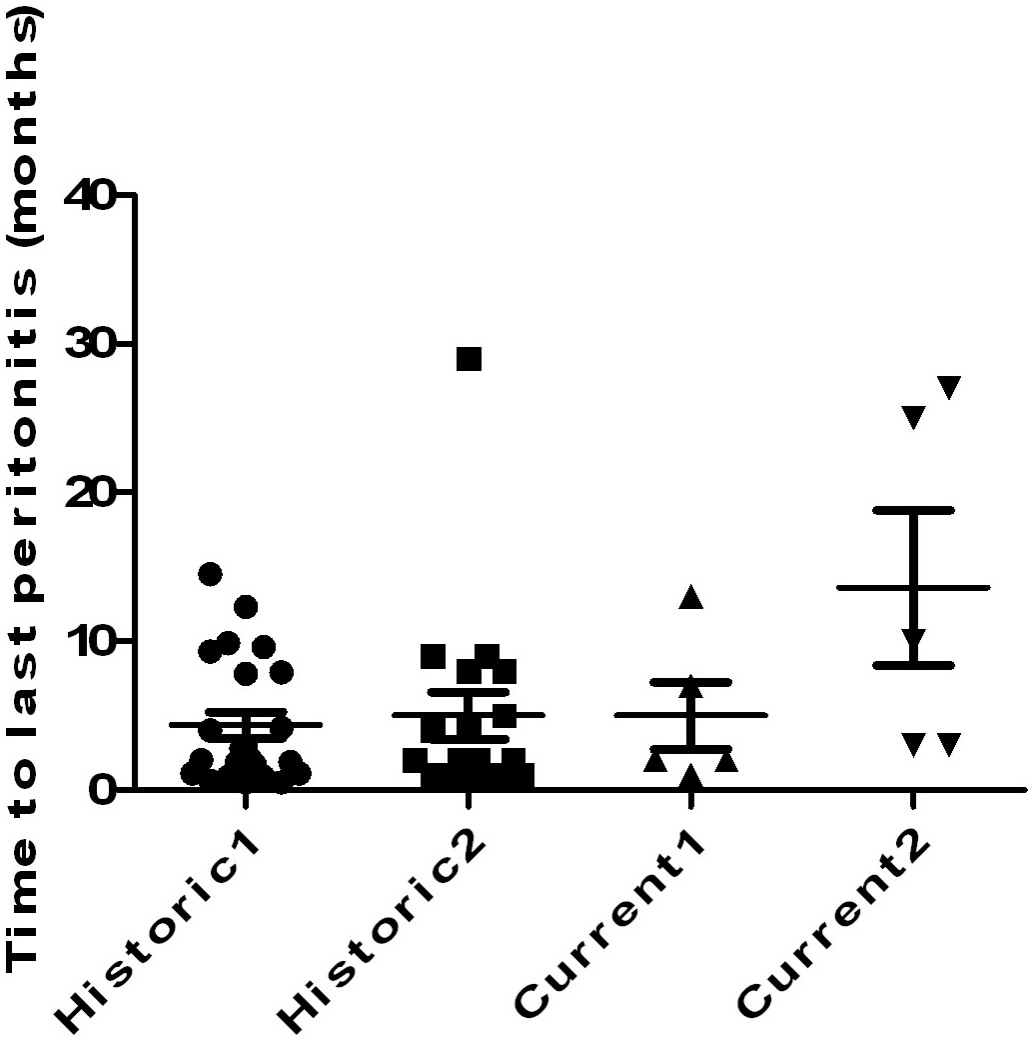
Diagram plotting the distribution of time between the last and current peritonitis episodes in different reports. Our contemporary cohort demonstrated a significantly longer latency compared with the others (*P*<0.01 between the after-2000 cohort and the historic 1 and 2 cohorts). Historic 1 cohort, data derived from *Am J Kidney Dis* 1989; 14(2): 101–4 Historic 2 cohort, data derived from *Perit Dial Int* 1994; 14(2): 174–7.

Table 2 shows the relevant features of the current *Acinetobacter* peritonitis episodes. The current peritonitis episode represented the first peritonitis episode in 62% of the patients, and for those who had already developed peritonitis, there were on average 2 episodes before the *Acinetobacter* episodes (Table 2). No significant difference in past peritonitis episode counts was observed between those that occurred before 2000 and those that occurred after 2000, but it was interesting to note that the time from the last peritonitis episode was borderline significantly longer in the after-2000 group (*P* = 0.07; Table 2). To verify this result, we further constructed a diagram comparing latency between the last peritonitis and *Acinetobacter* episodes within the historic cohorts and ours. We observed a temporal evolution of this feature, with lengthening of such latency (Figure 1; *P*<0.01).

**Table 2:** Features of *Acinetobacter* PD-associated peritonitis episodes.

*Clinical features*	*Total (n = 26)*	*Before 2000 (n = 12)*	*After 2000 (n = 14)*	*P value* *
**Parameters regarding previous episodes**				
Previous peritonitis episodesnumbers (if present)	2.1 (n = 10)	2 (n = 5)	2.2 (n = 5)	0.77
Time to last peritonitis(months)	9.3 (1–27)	5 (1–13)	13.6 (3–27)	0.05
Most recent PD peritonitispathogen	Culture-negative (6)Coagulase-negativestaphylococci (2)*Enterococcus fecalis*(1) MRSA (1)	Culture-negative(4) Coagulase-negativestaphylococci (1)	Culture-negative (2)Coagulase-negativestaphylococci(1)*Enterococcus* *fecalis* (1)MRSA (1)	
**Parameters of current episode**				
*Pathogen*				0.01
*Acinetobacter baumannii*	14 (54)	5 (42)	9 (64)	
*Acinetobacter iwoffii*	9 (35)	6 (50)	3 (21)	
*Acinetobacter ursingii*	1 (4)	0 (0)	1 (7)	
*Acinetobacter junii*	1 (4)	1 (8)	0 (0)	
Unspecified *Acinetobacter*	1 (4)	0 (0)	1 (7)	
*Presumed peritonitis origin*				<0.01
Break in exchange sterility	5 (19)	0 (0)	5 (36)	
Gastrointestinal bacterialtranslocation	5 (19)	1 (8)	4 (29)	
Exit site infection ortunnel infection	2 (8)	2 (16)	0 (0)	
Un-identified	14 (54)	9 (75)	5 (36)	
*Antibiotic susceptibilities*				
Aminoglycosides (gentamicin/amikacin)	**S** 26 (100)	**S** 26 (100)	**S** 26 (100)	
Ceftazidime	**S** 22 (84)	**S** 9 (76)	**S** 13 (93)	
	**I** 1 (4)	**I** 1 (8)	**I** 0 (0)	
	**R** 3 (12)	**R** 2 (16)	**R** 1 (7)	
Cefepime	**S** 26 (100)	**S** 26 (100)	**S** 26 (100)	
Ticarcillin	**S** 24 (92)	**S** 10 (84)	**S** 26 (100)	
	**I** 1 (4)	**I** 1 (8)		
	**R** 1 (4)	**R** 1 (8)		
Piperacillin	**S** 24 (92)	**S** 10 (84)	**S** 26 (100)	
	**I** 2 (8)	**I** 2 (16)		
Fluoroquinolone (ciprofloxacin,levofloxacin)	**S** 26 (100)	**S** 26 (100)	**S** 26 (100)	
Carbapenem	**S** 26 (100)	**S** 26 (100)	**S** 26 (100)	

*Comparison between before-2000 group and after-2000 group.

Continuous variables are expressed in mean (ranges), while categorical variables are expressed in number (percentage in parentheses).

Abbreviations: MRSA, methicillin-resistant *Staphylococcus aureus*; PD, peritoneal dialysis.

The presenting symptoms of PD-related peritonitis caused by *Acinetobacter* were abdominal pain (85%), followed by nausea/vomiting (31%), fever (27%), and diarrhoea (15%). All the patients exhibited a turbid dialysate. The mean effluent leukocyte count was 5296/µL (range, 148–13,300/µL), with 95% neutrophils (range, 90–98%). The initial blood leukocyte levels were 8094/µL (range, 3400–15,930/µL), with 82% neutrophils (range, 55–94%). The most common species from the effluent was *Acinetobacter baumannii* (54%), followed by *A. iwoffii* (35%), *A. ursingii* (4%), and *A. junii* (4%). *A. baumannii* was the predominant pathogen after 2000, while *A. iwoffii* outnumbered the others before 2000 (*P* = 0.01; Table 2). Two episodes (8%) showed polymicrobial growth (both involved *A. baumannii* and occurred after 2000). Coagulase-negative staphylococci and *Acremonium* species were each responsible for 1 episode of polymicrobial growth. None of the patients had concomitant *Acinetobacter* bacteremia.

The most common identifiable causes of PD-related peritonitis caused by *Acinetobacter* were a break in exchange sterility (19%) and translocation of GI microflora (19%), followed by exit site infection/tunnel infection (8%; Table 2). Approximately half of the episodes did not have an identifiable origin. It is interesting to speculate that an exchange sterility break emerged as the predominant origin of *Acinetobacter* peritonitis after 2000, while the proportions of causes related to exit site infection/tunnel infection and unidentified peritonitis declined significantly after 2000 (*P*<0.01).

The antibiotic susceptibilities of the *Acinetobacter* species are shown in Table 2. All the identified *Acinetobacter* species were susceptible to aminoglycosides (including gentamicin and amikacin), fluoroquinolones, and a fourth-generation cephalosporin (cefepime) after 1995. Approximately 16% of *Acinetobacter* species were non-susceptible to ceftazidime, among which three-fourths were resistant. In addition, 8% of isolates showed intermediate resistance to piperacillin, while 4% were resistant to ticarcillin. Intraperitoneal cefazolin/ceftazidime was initially used in 12 episodes (46%), and intraperitoneal cefazolin/aminoglycosides were used in another 12 episodes (46%; all before 1998). Two patients (8%) were initially treated with intravenous vancomycin/ceftazidime for septic shock. Antibiotics were shifted to intravenous ceftazidime and intraperitoneal gentamicin in 1 patient and intraperitoneal ceftazidime/gentamicin in the other after the patients stabilised with fair responses.

### Outcomes of Acinetobacter PD-associated peritonitis

Table 3 lists the clinical outcomes of the patients with PD-related peritonitis caused by *Acinetobacter*. A primary response was achieved in 16 episodes (62%), and after antibiotics, a secondary response was obtained in another 7 episodes (27%). There was no difference between time groups regarding antibiotic response percentage. The average time to antibiotic switch was 4 days (range, 2–9 days), and episodes that occurred after 2000 presented a significantly shorter time to antibiotic switch (*P*<0.01). Those responding to antibiotics were maintained on susceptible antibiotics for an average of 17 days (range, 12–21 days). About half of the episodes (46%) required hospitalisation, with more patients in the after-2000 group admitted (*P* = 0.01) but no significant difference in hospitalisation duration. A total of 3 patients (12%) with PD-related peritonitis caused by *Acinetobacter* had technique failure with catheter removal. The overall outcome was fair. None of the patients with *Acinetobacter* PD-associated peritonitis developed relapse or repeat peritonitis, and there was no mortality from such peritonitis.

**Table 3:** Outcomes of *Acinetobacter* PD-associated peritonitis.

*Variables*	*Total* *(n = 26)*	*Before 2000* *(n = 12)*	*After 2000 (n = 14)*	*P value* *
*Antibiotics*				
Primary response	16 (62)	7 (58)	9 (64)	0.76
Secondary response	7 (27)	3 (25)	4 (29)	0.62
Time to antibiotic switch(days)	4 (2–9)	6 (2–14)	3 (3–5)	<0.01
Total antibiotic duration(days)	17 (12–21)	16 (12–21)	18 (14–21)	0.28
*Hospitalization*	12 (46)	3 (25)	9 (64)	0.01
Length of stay	13 (5–23)	13 (10–14)	14 (5–23)	0.77
Tenckhoff catheter removal	3 (12)	2 (17)	1 (7)	0.47
Relapse peritonitis	0 (0)	0 (0)	0 (0)	
Repeat peritonitis	0 (0)	0 (0)	0 (0)	
Mortality	0 (0)	0 (0)	0 (0)	

*Comparison between before-2000 group and after-2000 group.

Continuous variables are expressed in mean (ranges), while categorical variables are expressed in number (percentage in parentheses).

Abbreviations: PD, peritoneal dialysis.

## Discussion

In the present study of *Acinetobacter* peritonitis episodes over more than 20 years, we found that *A. baumannii* was the most common species identified from the effluent, and the leading causes of infection were sterility break and GI microflora translocation. Nearly half required hospitalisation, but the outcome was good, with only 12% of technique failure and no mortality cases. In addition, temporal differences in the clinical features, including the interval between the last and current peritonitis episodes, increased significantly after 2000, along with more episodes from sterility break and less from exit site infection/tunnel infection. However, the fair outcome of *Acinetobacter* peritonitis surprisingly did not change with time.

Compared with past reports of *Acinetobacter* peritonitis, our patients (before 2000) were of similar age and carried a similar percentage of comorbidities (DM and autoimmune disorders; Tables 1 and 4). However, the mean age of the patients with *Acinetobacter* peritonitis seemed to be younger than the others with peritonitis from different pathogens (mean age: historic cohort, 41 years; our cohort, 52 years; ANZDATA, 61–64 years) [7],[10]. This might account for the fact that these patients manifested a milder comorbidity profile than the others with peritonitis (Table 1).

**Table 4:** Comparison between the current (after-2000) and the historic cohort.

*Characteristics*	*Historic 1* *(n = 23, 1989)* #	*Historic 2* *(n = 28, 1994)* &	*Current (after* *2000) (n = 14)*
Age (years)	41 (9–66)	NA	55 (23–84)
Gender (male %)	16 (70)	NA	7 (50)
**Comorbidities**			
DM	5 (22)	NA	6 (43)
Autoimmune disorders	2 (9)	NA	1 (7)
**Time to last peritonitis (months)**	4 (0.5–14.5)	5 (1–29)	13.6 (3–27)
**Pathogen**			
*Acinetobacter baumannii*	8 (35)	27 (96)	9 (64)
*Acinetobacter iwoffii*	3 (13)	1 (4)	3 (21)
*Acinetobacter ursingii*	0 (0)	0 (0)	1 (7)
*Unspecified Acinetobacter*	12 (52)	0 (0)	1 (7)
**Presumed peritonitis origin**			
Break in exchange sterility	4 (17)	NA	5 (36)
Gastrointestinal bacterial translocation	3 (13)		4 (29)
Exit site infection or tunnel infection	0 (0)		0 (0)
Un-identified	16 (70)		5 (36)
**Antibiotic susceptibilities**			
Aminoglycoside	**S** 20 (87)	NA	**S** 14 (100)
	**I** 3 (13)		
Ceftazidime	**S** 23 (100)	**S** 19 (69)	**S** 13 (93)
		**R** 9 (31)	**R** 1 (7)
Cefepime	NA	NA	**S** 14 (100)
Fluoroquinolone	**S** 23 (100)	**S** 22 (79)	**S** 14 (100)
		**R** 6 (21)	
**Outcomes**			
Tenckhoff catheter removal	2 (9)	10 (36)	1 (7)
Recurrent peritonitis	1 (4)	0 (0)	0 (0)
Mortality	0 (0)	1 (4)	0 (0)

NA, not available.

# Adapted from Galvao C et al. *Am J Kidney Dis* 1989; 14(2): 101–4.

& Adapted from Lye WC et al. *Perit Dial Int* 1994; 14(2): 174–7.

*Acinetobacter* species are strictly aerobic, pleomorphic, and non-motile GNB. To date, 26 named species and 9 genomic species have been identified, among which *A. baumannii* and *Acinetobacter* genomic species 3 and 13TU are collectively referred to as the *A. baumannii* complex [22], [23]. Towner and colleagues identified 3 major overlapping populations of *Acinetobacter* species, each with different epidemiologic and microbiologic features [24]. First, *A. baumannii* complex species are prone to develop antibiotic resistance and frequently cause nosocomial outbreaks through their persistent presence in health-care facilities. Second, *A. iwoffii* and *A. johnsonii* are antibiotic-susceptible skin commensal flora of animals and humans, mostly resulting in food spoilage. Finally, *A. calcoaceticus* and other *Acinetobacter* members are ubiquitous colonisers in soil and wastewater, with low virulence and high antibiotic sensitivity. In our PD peritonitis series, the predominant pathogen differed with time; *A. iwoffii* was the predominant offender before 2000 but was replaced by *A. baumannii* after 2000 (Table 2). This seems to suggest that the ecology of *Acinetobacter* breaching the peritoneum has changed temporally, from common skin flora (*A. iwoffii*) to colonisers of health-care settings (*A. baumannii*). This transition might reflect the improvement of exchange systems and increased utilisation of health-care resources as time passed.

Galvao *et al.* proposed that *Acinetobacter* PD peritonitis often occurred during a “vulnerable period, when hosts were immunocompromised,” especially in the months immediately following another PD peritonitis episode [12]. This theory was subsequently embraced by other researchers, and the average period between the last peritonitis and *Acinetobacter* episodes was 4 to 5 months at that time (Table 4) [13], . However, from our findings (Figure 1), the formerly recognised susceptible period of developing *Acinetobacter* peritonitis seemed vanishing in the modern PD era, and now most episodes did not follow the previous peritonitis as closely as they did in the past. Furthermore, the proportion of PD peritonitis cases from sterility breaks in our cohort also increased significantly after 2000, with a decrease in peritonitis originating from unidentified sources (Tables 2 and 4). We proposed that the development of *Acinetobacter* peritonitis in the modern era might not result from a dysregulated host immune status in PD patients (as before), but more from the hygiene breaks and failure to perform sterile exchange procedures. This phenomenon could be reminiscent of the routes of staphylococci infections.

The choice of treatment for *Acinetobacter* PD-associated peritonitis is contrary to what we might expect if extrapolating from the experiences of hospital-acquired *Acinetobacter* species infections [25]. In our cohort, all isolates were susceptible to aminoglycosides, fourth-generation cephalosporins (available after 1995), and fluoroquinolones, whilst only 10% to 20% were non-susceptible to third-generation cephalosporins or anti-pseudomonal penicillins (Table 2). However, strains with resistance to aminoglycosides or fluoroquinolones did exist (Table 4). Based on the findings of past reports and ours, we propose that fourth-generation cephalosporins might be a more suitable choice for *Acinetobacter* PD peritonitis, followed by aminoglycosides or fluoroquinolones. Empirical use of ceftazidime might carry a 10% to 30% risk for treatment failure.

An interesting issue among our findings is that the outcomes of *Acinetobacter* peritonitis remained fair throughout the follow-up period, despite the increase in *A. baumannii* percentage. *A. baumannii* infections are reportedly associated with 10% to 20% higher attributable mortality and longer length of hospital stay [2], [26], [27], and inappropriate antibiotic regimens further increase the risk [28]. Thus, the observed fair outcome in this series of predominantly *A. baumannii* PD-associated peritonitis is unusual. We proposed several reasons for this finding. First, the overall antibiotic resistance rate in our *Acinetobacter* effluent isolates is quite low (including *A. baumannii*), and this phenomenon holds true in other major PD peritonitis series as well (Table 4) [8], [12], [14]. Second, the empirical antibiotic regimens recommended by the ISPD for peritonitis invariably contain aminoglycosides or ceftazidime, both with fair efficacy against *Acinetobacter* isolates [5], [20], [21]. Consequently, the chance of treatment failure would be expectedly low; therefore, clinical outcomes would be better in this specific type of *Acinetobacter* infection than in the other types. Finally, as we explained, the origin of *Acinetobacter* PD peritonitis has gradually shifted from poor host immunity to sterility break. This might also play a role in the relatively fair outcome of *Acinetobacter* peritonitis.

To our knowledge, this study is the most up-to-date and comprehensive report of PD-associated peritonitis caused by *Acinetobacter*, with focused descriptions of their clinical features. The temporal changes in the characteristics of patients with *Acinetobacter* peritonitis include an increasing proportion of *A. baumannii*, more cases from breaks in exchange sterility, fewer cases from catheter infections, and an increasing interval between the previous and the present peritonitis episodes. These features might have important epidemiologic meanings in the PD field and warrant our continuous attention.
